# Marked long-term decline in ambient CO mixing ratio in SE England, 1997–2014: evidence of policy success in improving air quality

**DOI:** 10.1038/srep25661

**Published:** 2016-05-23

**Authors:** D. Lowry, M. E. Lanoisellé, R. E. Fisher, M. Martin, C. M. R. Fowler, J. L. France, I. Y. Hernández-Paniagua, P. C. Novelli, S. Sriskantharajah, P. O’Brien, N. D. Rata, C. W. Holmes, Z. L. Fleming, K. C. Clemitshaw, G. Zazzeri, M. Pommier, C. A. McLinden, E. G. Nisbet

**Affiliations:** 1 Dept. of Earth Sciences, Royal Holloway, Univ. of London, Egham, Surrey, TW20 0EX, United Kingdom; 2 Institute of Technology in Architecture, ETH, CH-8093 Hönggerberg, Zürich, Switzerland; 3 Darwin College, Cambridge, CB3 9EU, UK; 4 School of Environmental Sciences, University of East Anglia, Norwich NR4 7TJ, UK; 5 Escuela de Ingeniería y Ciencias, Tecnologico de Monterrey, Campus Monterrey, Av. Eugenio Garza Sada 2501, Monterrey, N.L., México, 64849; 6 NOAA/ESRL Global Monitoring Division, 325 Broadway GMD-1, Boulder CO 80303, USA; 7 Irish Environmental Protection Agency, Richview, Clonskeagh Rd., Dublin 14, Ireland; 8 St. Richard’s Church, Forge Lane, Hanworth, Middlesex, TW13 6UN, UK; 9 National Centre for Atmospheric Science (NCAS), Department of Chemistry, University of Leicester, Leicester, LE1 7RH, UK; 10 Sorbonne Universités, UPMC Univ. Paris 06, Université Versailles St-Quentin; UMR8190, CNRS/INSU, LATMOS-IPSL, Paris, France; 11 Environment and Climate Change Canada, Air Quality Research Division, Toronto, Ontario MTH 5T4, Canada

## Abstract

Atmospheric CO at Egham in SE England has shown a marked and progressive decline since 1997, following adoption of strict controls on emissions. The Egham site is uniquely positioned to allow both assessment and comparison of ‘clean Atlantic background’ air and CO-enriched air downwind from the London conurbation. The decline is strongest (approximately 50 ppb per year) in the 1997–2003 period but continues post 2003. A ‘local CO increment’ can be identified as the residual after subtraction of contemporary background Atlantic CO mixing ratios from measured values at Egham. This increment, which is primarily from regional sources (during anticyclonic or northerly winds) or from the European continent (with easterly air mass origins), has significant seasonality, but overall has declined steadily since 1997. On many days of the year CO measured at Egham is now not far above Atlantic background levels measured at Mace Head (Ireland). The results are consistent with MOPITT satellite observations and ‘bottom-up’ inventory results. Comparison with urban and regional background CO mixing ratios in Hong Kong demonstrates the importance of regional, as opposed to local reduction of CO emission. The Egham record implies that controls on emissions subsequent to legislation have been extremely successful in the UK.

Here we present continuous records of atmospheric measurement of carbon monoxide (CO) at the Queen’s Building, Royal Holloway, University of London, near the town of Egham. The Egham (EGH) long-term record is unique in the UK in its unbroken length of high precision measurement, and in its setting at the boundary of a densely populated urban area with exposure both to polluted urban air as well as for comparison to clean unpolluted background air masses.

Emissions of CO to the atmosphere come from a range of sources, but mostly as a by-product of inefficient combustion, whether it is biomass burning, domestic heating systems or vehicle exhausts. The lifetime of a CO molecule in the atmosphere with respect to destruction by OH varies strongly with latitude, from ten days in summer continental regions and less than a month in the equinoctial tropics, to nearly a year in high northern latitudes^1^. Thus while intercontinental transport does occur (especially in winter), high CO tends to be measured close to source. As such it is a good target for regional emissions control.

Novelli *et al*.^2^ reported a sustained decrease in tropospheric CO during the 1990s at a rate of about 0.5 ppb yr^−1^, although there were shorter periods of increase and decrease. This trend had begun to reverse a sustained 1–2 ppb yr^−1^ growth during the 1980s. The 1990s decline was largely driven by Northern Hemisphere change. This decline has been sustained as both satellite and ground-based measurements report declining global CO^3^,^4^,^5^,^6^,^7^.

In the United Kingdom (UK), effective emission control for CO dates from the response to the ‘Great Smog’ of 1952, which may have led to an excess mortality of about 12,000 deaths^8^. The consequent Clean Air Acts of 1956 and 1968 eliminated domestic coal burning in London. Moreover during the 1970s and 1980s there was widespread conversion of UK domestic heating from coal fires to hot water systems using natural gas. CO is a useful proxy for assessing reduction in vehicle emissions since the introduction of the 3-way catalytic converter for petrol vehicles and catalytic diesel exhaust oxidation. However, it was only in the 1990s that vehicle emissions were properly addressed when in 1991 the Road Vehicles (Construction and Use) Regulations introduced better control on new vehicle emissions, and was followed by the Environment Act of 1995. In 1997 the UK National Air Quality Strategy^9^ introduced rigorous annual vehicle exhaust testing.

Although CO measurement has been widespread in SE England^10^ most measurements are for local authority monitoring purposes, and in meteorologically unrepresentative sites. These measurements are to Automatic Urban and Rural Network (AURN) and London Air Quality Network (LAQN) standards of QA and QC: all (NDIR) instruments are routinely calibrated and every 6 months are fully serviced and undergo an intercalibration audit. However, they are not calibrated against NOAA (WMO) standards^11^,^12^, and of lower precision and accuracy than attained for NOAA background measurements. Sites can be purposefully subject to very local road emissions or perturbations in sources (e.g. new buildings, or traffic flow adjustments).

Arguably the best suited inner London site for long-term urban background study was the record from Bloomsbury in central London^10^, which was monitored from 23 January 1992 until 16 July 2012, when it was discontinued. A continuing urban record is the long-term site at Marylebone Road, central London^10^,^11^. This site is on a very busy road (‘street canyon’) on the boundary of the central London ‘congestion charge’ area where heavy vehicle entry fees were introduced in 2003. Therefore this site is influenced by very local emissions and is not representative of wider SE England. However, for the Marylebone Rd. site, a remarkable and sustained 12% per year drop in CO has been reported over the 1998–2009 period^13^. This record implies that CO in central London has declined markedly in the past 15 years, and von Schneidemesser *et al*.^13^ report data from a range of sites suggesting this trend is widespread.

Bigi and Harrison^12^ reported CO mole fraction measurements in N. Kensington, central London, in the 2001–2008 period. CO shows a typical traffic-associated pattern. There are two daily peaks, with lower abundances at the weekend, and annual minima in June and July. Overall in the 2001–2008 period they found a clear downward trend in CO. Comparable urban results have been found in Valencia, Spain, where Capilla^14^ reported a decrease in CO between January 1994 and December 2004, with 12-month decline rates varying between 10.5% and 17.6%, much higher than for European continental background^15^.

Our high-precision data reported here cover the full period since the National Air Quality Strategy was implemented, and clearly show the results: there has been sustained improvement. Here we assess these improvements as observed in the EGH 1997–2014 record in context of the Mace Head, Ireland, background time series and the central London roadside Marylebone Road time series. The changes in the London region are compared with Hong Kong, a city area of similar size, to consider how measured mixing ratios should be changing in future given regulations now in place.

## Geographical Setting and Meteorology

**Figure 1:**
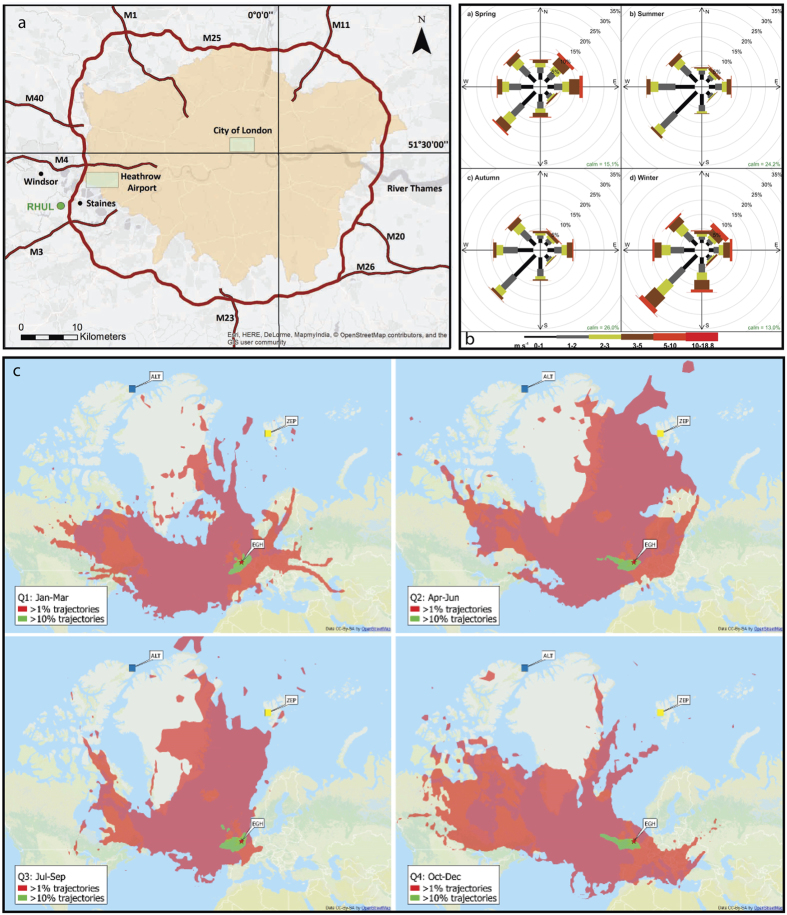
Background and meteorology for the Egham (EGH) site. (**a**) Royal Holloway Univ. London (RHUL – green) is located WSW of central London. Atlantic air typically crosses the UK south coast and then descends over large tracts of agricultural or wooded land and scattered towns to reach Egham. Image created in ArcGIS ArcMap 10.2 using basemap source: Esri, HERE, DeLorme, MapmyIndia. © OpenStreetMap contributors, and the GIS user community. (**b**) Wind directions for air arriving at Egham (EGH), 2000–2014, by percentage, season and wind speed. Note the dominance of winds from the western sectors. (**c**) HYSPLIT 4 frequency plots of 10 day back trajectories from Egham for 2011 to give an indication of annual variability of the air mass origins^16^,^17^. Each panel represents 3 months of data, with a new trajectory plotted every 6 hours. Quarter 1 (Jan–Mar), Q2 (Apr–Jun), Q3 (Jul–Sep), Q4 (Oct–Dec). Red star is EGH. Lime green indicates >10% of trajectories and pale red >1% of trajectories. Air is mainly from Canada, Europe and the Arctic, with very little air from high CO regions in east Asia and the USA. Note for 2011 the greater incidence of easterly trajectories in Q4 autumn and the dominance of south-westerlies in Q1 winter. For ease of interpretation, some Arctic background sites (squares on the maps) have been added: Zeppelin (Ny-Ålesund) is yellow, Alert is dark blue. HYSPLIT models are produced by ARL (Air Resources Laboratory). HYSPLIT.trajectory maps are produced using archive data and can be freely redistributed (https://www.ready.noaa.gov/HYSPLIT_agreement.php).

The Royal Holloway Greenhouse Gas Laboratory (details in Supplementary Information S1) is on a hillside 32 km WSW of central London (51°25′36″N, 0°25′40″W) (Fig. 1a). In contrast to urban street-side measurements, the long-term high-quality EGH record of ambient air, well away from extreme local anomalies, provides a basis for more general assessment of the overall regional CO trend.

The EGH site is ideally situated close to the boundary of the London conurbation (Fig. 1a), with green belt and large wooded tracts between the SSW and WSW. It experiences frequently varying airflows, bringing in both clean air and polluted. The prevailing airflows are SW, from the Atlantic (Fig. 1b). Hysplit trajectory analysis^16^,^17^ during periods of prevailing airflow from the Atlantic (Fig. 1c) indicates that typical air destined for Egham approaches the UK coast along the English Channel, coming ashore at altitude and then descending over mainly semi-rural land. Although there are some coastal cities under typical trajectories, only in the last few km does the air approach local sources. Figure 1c shows the large mix of air mass sources arriving at the Egham site during an annual cycle. The dominance of SW and SSW prevailing winds over London was first reported in 1833 by Howard^18^. Atlantic air dominates in all seasons, but in autumn (October-December) and winter (Jan-March) Egham receives frequent easterlies that have crossed the conurbation.

East of Egham is the London basin (Fig. 1a), heavily populated and including the Greater London urban area. The meteorological boundary layer can be sometimes as low as 100 m, when the nocturnal inversion is low near dawn. Calm anticyclonic conditions occur on multiple occasions through the year. Although wind speeds are usually very low in anticyclonic conditions (<1 ms^−1^), there are frequent episodes when the London heat island produces overnight breezes (comparable to land and sea breezes) under the inversion, bringing air from the warm core of the urban area. Thus the RHUL measurement location at Egham is well placed: it experiences both relatively clean Atlantic background air, and broadly samples integrated regional polluted air from the densely populated London basin.

## Results

### Overall Synopsis of Results

Over the observation period there has been an overall downward trend in the annual average of the ambient mixing ratio of CO in Egham air (Table 1). When the Atlantic background CO is subtracted, the reduction is attributable to lower regional emissions in SE England and nearby regions.

**Figure 2:**
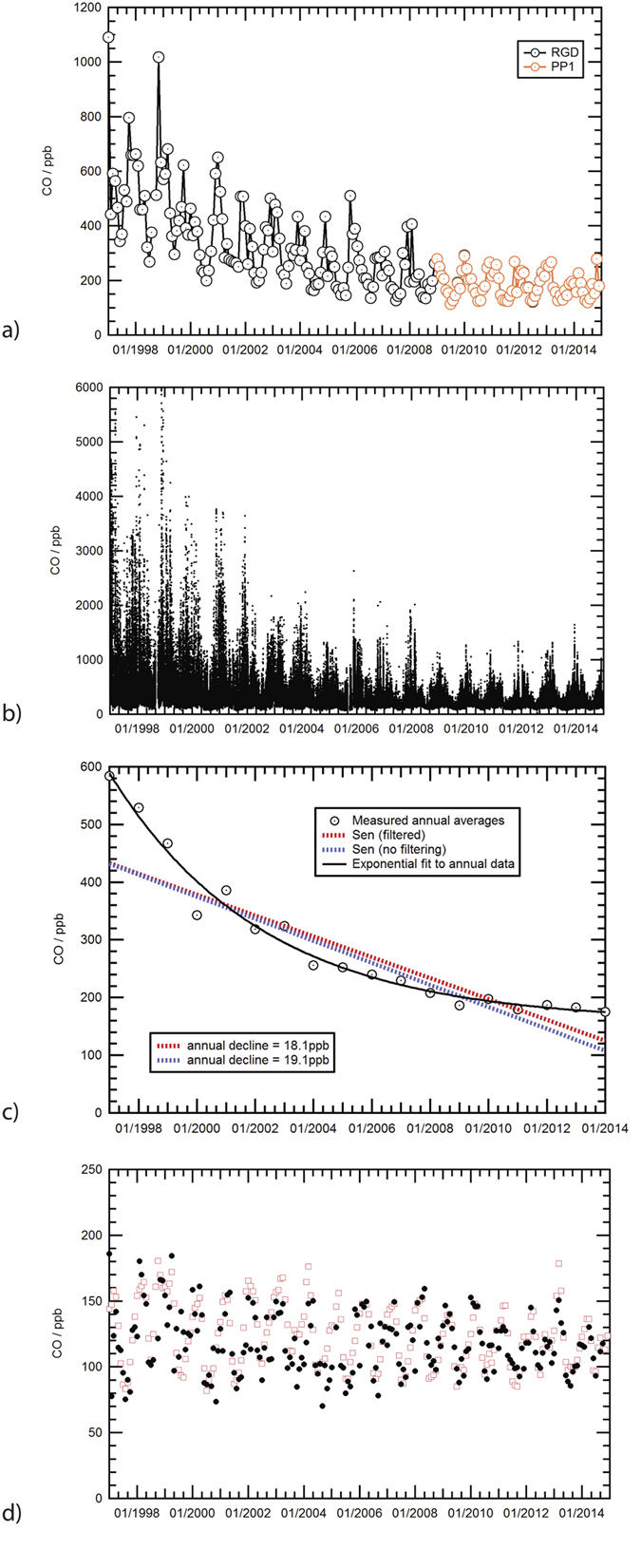
CO trends measured at the EGH site. (**a**) The overall London (Egham) CO Record from 1997–2014. Circles show average monthly mixing ratio (in ppb). Analysed by Trace Analytical RGD-2 instrument (black circles) prior to 2009, and Peak Performer 1 analyser thereafter (orange circles). (**b**) Daily averaged mixing ratios during Jan 1997-Dec 2014 of CO recorded at Royal Holloway following the format described by Hernandez-Panagua *et al*.^19^. (**c**) Linear trends of CO at the EGH site during 1997–2014 calculated with the Sen’s estimate for annual averages and secular trend. The secular trend was obtained by filtering monthly averages with the STL technique^20^. The best fit to the data is an Exponential curve to the annual CO average (black dashed curve) using an offset exponential function in the form: 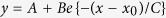 where A = 159.26, B = 430.25 and C = 5.0887, and x_0_ is the initial year of measurements, 1997. (**d**) Comparison of filtered Egham background CO (black solid circles) vs. Mace Head monthly averages (red open squares), 1997–2014.

Figure 2a shows the 1997–2014 monthly average mixing ratios for CO. The overall decline in annual average values is dramatic. The 1990s had very much higher CO than post-2000 with a decline rate faster in the earlier years, e.g. −64 ppb in 1998. The reduction since 2000 is slower, but with sustained decline in CO until 2009 since when the minor fluctuations are predominantly a function of local meteorology. The overall trend in the daily data is the same (Fig. 2b). In particular, the very high daily maxima, especially in winter episodes of stagnant air under a low inversion, characteristic of the late 1990s, are now absent. The weekly averages (not shown here) display a similar pattern, implying there were far fewer sustained high-CO events in recent years than in earlier years. Thus both daily peaks and sustained anticyclonic high-CO episodes are greatly reduced. Fig. 2c shows that the annual year-on-year change averages 18 ppb/yr, calculated using the Sen’s estimate^19^. The secular trend was obtained by filtering monthly averages with the STL technique^20^. The change is best described by a curve fit using an offset exponential function (Fig. 2c), demonstrating how the rate of decline has slowed in a quasi-linear fashion in recent years as values approach those typical of the Atlantic background.

There are three possible causes of declining CO mixing ratios: 1) a decline in the Atlantic background; 2) a decline in local sources in London and SE England, and 3) a decline in continental European sources. As the Egham CO record includes both frequent background episodes of SSW-SW Atlantic air, and episodic easterly, polluted air, it is necessary to separate these two wind direction components, in order to demonstrate whether the decline is dominated by a reduction of global or local sources.

### Comparison Between Atlantic and Egham ‘Background’ Air

The term ‘Background air’ is here taken to denote regional air masses entering the area of study. Background CO is strongly seasonal. The principal source of London’s background is Atlantic air, but many air masses arrive from the European continent.

CO data were filtered, considering all measurements, for wind speeds >1 ms^−1^ with CO *less* than 15 ppb above the contemporary monthly minimum (see Supplementary Information S2 for details). This simple filter, based on practical experience, was adopted to permit variations in Atlantic background air, while the 1 ms^−1^ cut-off excludes very local air masses with high inputs from nearby CO sources. A more generalised mathematical filter would risk missing the rapidly shifting event-by-event texture of the Atlantic meteorological background. After this removal of the “contemporarily-high CO” data, the monthly average of the remaining population of measurements was taken as the “Egham background”.

These results were then compared with the monthly averages for “clean” regional Atlantic background air as defined by the 1997–2014 measurements of NOAA flasks^21^ at Mace Head, Ireland (Fig. 2d). Two inferences can be drawn from this comparison. First, although the ‘background’ air episodes are sporadic (especially in the earlier part of the 1997–2014 period), Egham does indeed experience significant periods when the air is effectively ‘Atlantic background’ quality, and has experienced such episodes ever since 1997. Secondly, Egham background values throughout the 1997–2014 period are increasingly comparable to the Mace Head record. In recent years, as overall Egham CO has fallen, this comparability has improved. Indeed, a few Egham measurements are occasionally below the contemporary Mace Head clean-sector NOAA flask values. This would be expected as Mace Head is further north on the Coriolis curve of the Atlantic westerlies and often samples air from Canada, while in contrast clean-sector trajectories arriving at Egham via the English Channel are often from the mid-Atlantic.

Given its broad comparability with the ‘Egham background’, the Mace Head record has been taken as background throughout the 1997–2014 analysis, to provide a better sustained basis of comparison. Smoothing is deliberately *not* applied to the data set as this would introduce erroneous deviation from actual winds, delivered in the changing event-by-event meteorology, where significant real changes can occur rapidly.

### NAME Trajectory Analysis of Regional Background Air

**Figure 3:**
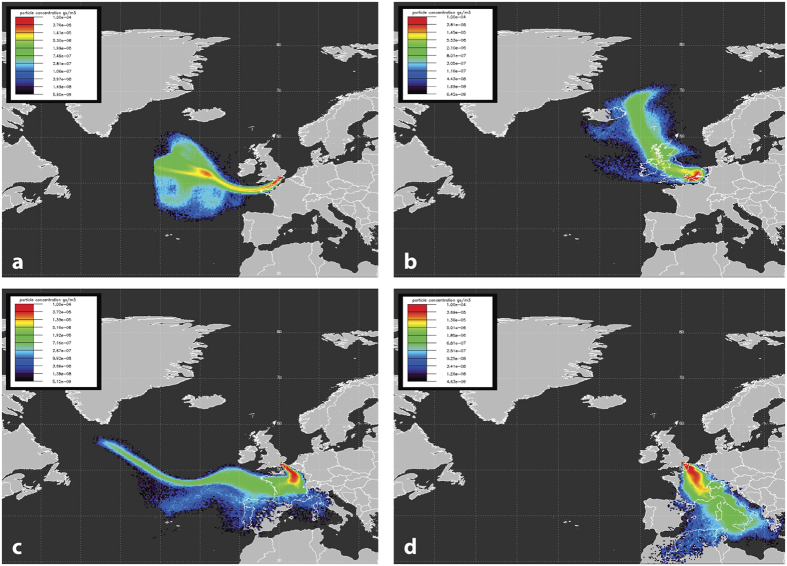
NAME particle dispersion modelling of air masses reaching the EGH site. Scenarios run using version 6.1 of the Met Office NAME particle dispersion model^22^ (http://www.metoffice.gov.uk/research/modelling-systems/dispersion-model) to allow back trajectory analysis of certain events during the measurement period. © British Crown copyright 2016, Met Office. Plots show the surface influence (0 to 100 m) in the preceding 5 days. All plots are for 0300 UTC because the time for lowest influence of local area combustion sources is between 0200 and 0400 and mixing ratios shown below are averages of this 2-hour period. Two events are highlighted from July 2001 (**a**,**b**) and November 2011 (**c**,**d**). (**a**) Low CO (104 ppb) representing Atlantic background on 18 July 2001, (**b**) higher CO of 224 ppb from the addition of UK and near continental emissions on the previous day 17 July 2001, (**c**) Atlantic air with added emissions from France and SE England reaches 228 ppb on 19 November 2011, (**d**) 3 days later on 22 November 2011 the 5 days of air movement crosses Europe from the Mediterranean Sea and reaches 366 ppb CO. During a still-air period of this event at 00:00 UTC on 21/11/2011 the CO reached a high of 1257 ppb due to emissions of local CO sources under the inversion.

The UK Meteorological Office Numerical Atmospheric-dispersion Modelling Environment (NAME)^22^ was used to investigate origins of air masses arriving at Egham with both near-background and higher CO contents. The example 5-day backward surface level air mass dispersion maps (footprints) shown in Fig. 3 are from 2001 and 2011. These show the likelihood of air arriving from a particular direction at times that background and higher CO mixing ratios are measured at the EGH site. Background mixing ratios of 90–120 ppb have changed little over the measurement period and even as far back as July 1998 there was a period of near-comparable mixing ratios at Mace Head and Egham that lasted approximately 3 days, demonstrating that during periods of ‘background air masses’ with South-Westerly winds (Fig. 3a) the air being sampled at the Egham and Mace Head sites is essentially the same. Local sources dominate around Egham, but when only wind speeds >1 m/s are selected, any local combustion sources have largely dispersed by the middle of the night so that the daily minima, representing the wider regional source increment, is recorded between 0200 and 0400 local time. Although mixing ratios of >1200 ppb were still rarely being recorded during winter events in 2011, the local sources made up the majority of the excess, with UK sources adding around 100 ppb (Fig. 3b) and continental sources up to 250 ppb (Fig. 3c,d) increments to the background.

### The ‘Egham Residual’: Assessment by Wind Direction

**Figure 4:**
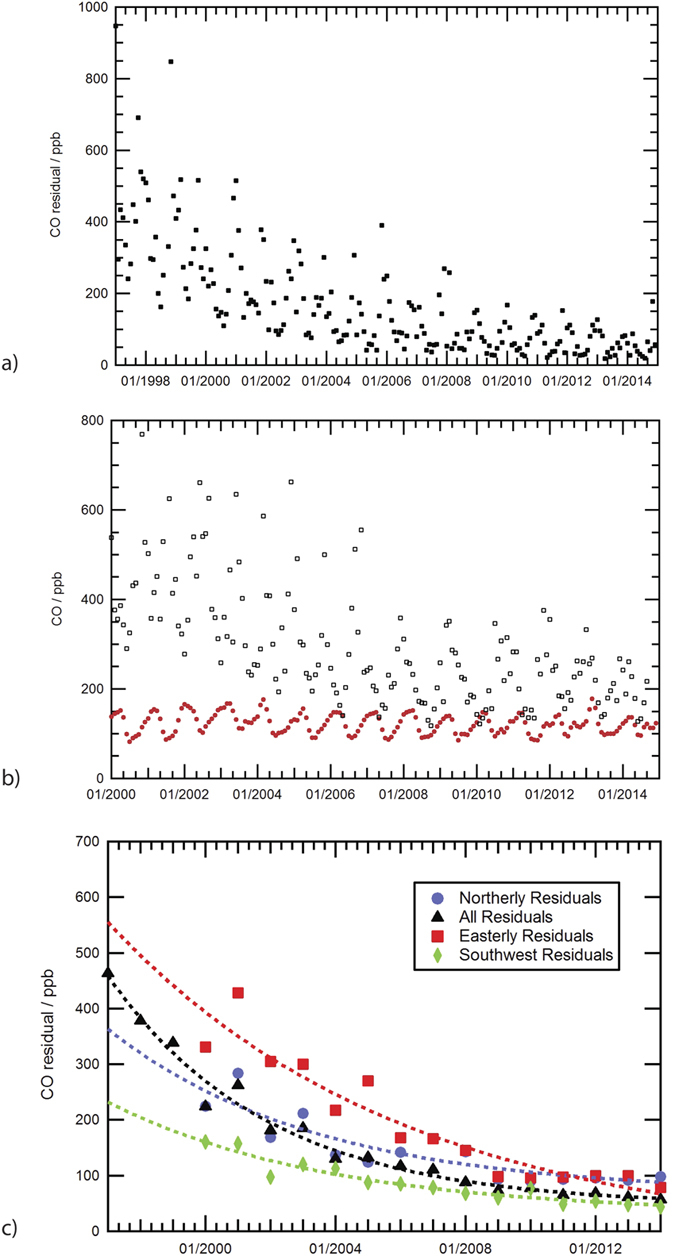
Assessment of CO reductions at EGH compared to background and by wind direction. (**a**) Monthly averaged residual CO increment at Egham for all data, after subtracting the Mace Head background. (**b**) Comparison of monthly averaged CO in easterly winds (>0.1 m/s) arriving at Egham (Black open squares) compared to the Mace Head monthly-averaged Atlantic background values, (Red solid circles). (**c**) Directional analysis of the ‘Egham residual’; the CO increment over Mace Head background mixing ratios. Black curve: All directions. Blue curve: Northerly winds arriving from the northern UK and North Sea; Red curve: Easterly winds only: air from London and NW continental Europe; Green curve: SW winds from Atlantic background sector.

The ‘Egham (London) residual’ is here defined as the excess CO in London air (as measured at Egham) compared to the contemporary NOAA Mace Head background: i.e. the residual value when contemporary monthly averaged Mace Head CO is subtracted from Egham CO. In the late 1990s, typical monthly averaged ‘Egham residual’ CO values were 250–550 ppb (Fig. 4a). There were frequent episodes when daily CO maxima at Egham far exceeded 1000 ppb (though note the Reduction Gas Detector (RGD) instrument then in use was not linear at high CO mixing ratio: see Supplementary Information S1). Values reaching 10,000 ppb were measured occasionally under low early morning inversions in sustained winter anticyclones, with one month in 1997 when even the monthly averaged residual was near 850 ppb CO. By 2011, typical daily averages were within 100 ppb of Atlantic background values, and in moving air, especially in summer, the Egham measurements were frequently within 20 ppb of background Atlantic values.

Figure 4b shows the importance of easterly winds in bringing high CO air to Egham. East of Egham lies most of the London basin, and east of that, NW Europe. Thus the CO in winds from the easterly sector tracks the broad history of CO in NW Europe. CO in these winds shows a sharp decline since 1999, suggesting that emissions have been strongly reduced not only in the UK but in much of nearby NW Europe.

Figure 4c shows the annual average Egham residuals over Mace Head from specific directions (45° sectors), with fitted exponents (note that complete Egham meteorological data is available only from 2000 onwards). The black curve demonstrates the general decline in CO from all wind directions. Red shows the Easterly sector, which includes CO blown over from London and continental Europe and has the highest residuals declining from 400 ppb in 2000 to 70 ppb in 2014. The Northerly sector winds that bring air from the Arctic via East Anglia and Northern England, and from the North Sea oil and gas fields now has the highest residual. The decline from 210 ppb to 80 ppb is more modest but still clear. The SW sector air is closest to Atlantic background air, with a residual decreasing from 130 ppb in 2000 to 40 ppb in 2013–14. Overall, in all sectors the decline is consistent with sustained reduction in emissions where larger sources are eliminated first, and thereafter reduction becomes increasingly difficult.

### Comparison with Inner London

**Figure 5:**
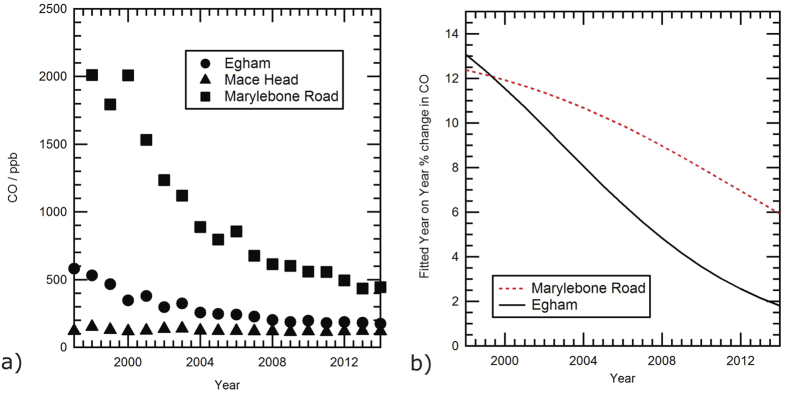
CO trend comparisons between background, peri-urban and city roadside sites. (**a**) Comparison between CO annual averages from Marylebone Rd.^11^, Egham (this work), and Mace Head^21^. (**b**) Exponentially fitted year-on-year change from annual averaged CO at Egham and Marylebone Rd.

There is a multi-year CO record from Marylebone Rd. in central London^10^,^11^. The instruments measure in 0.1 ppm intervals so the precision is 1–2 orders of magnitude lower that the ±1–2 ppb for data recorded at EGH, as the measurements are designed to monitor health-related air quality compliance, but this is sufficient at a very polluted site, and enough for comparison of the relative rates of CO decline. Marylebone Road is a very busy urban street canyon on the northern edge of the inner London congestion charge area and therefore receives much greater local emissions compared with the semi-rural site of Egham. As mentioned above, von Schneidemesser *et al*.^13^ report a sustained 12% per year drop in urban CO in central London over the 1998–2009 period. This is comparable to the decline rates reported in Valencia, Spain^14^, although in London more recent data from Marylebone Road suggests that the rate of decline is now starting to drop (Fig. 5a). Both at Egham and Marylebone Road there has been a sustained decline in average CO, but the rate of decline has been much faster at the Marylebone Road.site than at Egham (Fig. 5b), implying measures to control vehicle and therefore central London CO emissions have been effective.

On 17 February 2003 the London Congestion Charge area was introduced, making car entry into central London expensive (currently in 2016, £11.50 (US $17) per day). Since the charge was introduced, central London has had fewer light vehicles (e.g. cars) but a higher proportion of taxis and buses. Electric and hybrid vehicles are exempt from the charge. Marylebone Road is just outside the Congestion Charge area and marks the area’s northern boundary. In the two decades from 1994 to 2013 the percentage of diesel cars rose from 7.4 to 34.5% of those on the road and currently account for 50% of the annual 2 million new car sales in the UK^23^. Diesel cars on average emit only 6.5% of the CO from the averaged petrol car^23^, so this change will have accelerated the decline in CO emissions from areas dominated by diesel powered vehicles, such as central London or the orbital motorway near EGH.

### Satellite Observation

Remote sensing from space confirms the inferences from the Egham record. Pommier *et al*.^24^ report an application over megacities of the new CO retrieval algorithm applied to “Measurements of Pollution in the Troposphere” (MOPITT) satellite data (version 5) that combines the thermal infrared (TIR) with near-infrared (NIR) bands that are more sensitive to the boundary layer. We also used European Centre for Medium-term Weather Forecasting (ECMWF) reanalysis wind data, averaged to encompass the boundary layer from the surface to 800 hPa. The wind fields are then interpolated spatially and temporally to the location and overpass time of each MOPITT pixel. The method subtracts upwind from downwind values, to extract the urban CO increment. The distance of the maximum value from the urban centre depends on the wind speed: in calm conditions, this maximum will be close to the core of the city, while in high winds the maximum CO will be significantly on the downwind flank of the urban core. The results show a clear reduction of CO over most studied sites.

**Figure 6:**
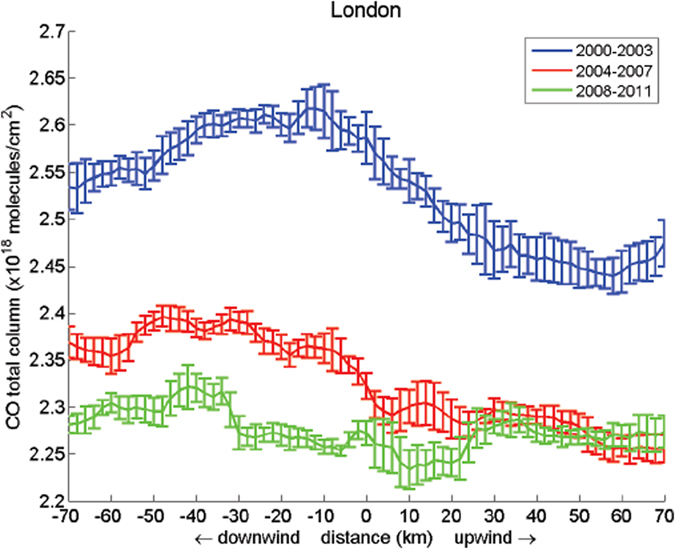
Satellite analysis of CO reductions over London. MOPITT total column mean for CO, zonally integrated (±10 km) for each 2 km after repositioning of all satellite observations about the city centre in order to co-align all wind vectors (rotation of all pixels) in an upwind-downwind direction at the time of observation over London, as a function of the distance from the city centre, for 2000–2003 (blue), 2004–2007 (red) and 2008–2011 (green). Wind data has been averaged to encompass the boundary layer from the surface up to 800 hPa. The error bars correspond to a standard deviation. Note sustained reduction in CO, sharper between 2000–2003 and 2004–2007 than between 2004–2007 and 2008–2011. Data were obtained from the NASA Langley Research Center Atmospheric Science Data Center (ftp://l5eil01.larc.nasa.gov/MOPITT/).

A high-resolution assessment of the CO distribution over London is shown in Fig. 6. The downwind – upwind contrast shows the urban increment. Three time segments are shown. A sustained overall reduction in CO between 2000–2003, 2004–2007 and 2008–2011 is observed, especially in the core of the urban area between the first two time segments. This estimate of the decline is imprecise as it is very sensitive to the arbitrary choice of boundary height for the winds, but broadly implies the reduction has been ongoing in the post-2000 period. Overall, the finding is consistent with the *in situ* evidence from the Egham record.

### Regional Context and Comparison with Emissions Inventories

The CO mixing ratio is being reduced both globally and also regionally in the European Union. As mentioned above, von Schneidemesser *et al*.^13^ report a sustained drop in urban CO in central London over the 1998–2009 period. Regionally in central Europe, Zellweger *et al*.^15^ observed a sustained drop of −2.65 ± 0.04 ppb/yr from 1991–2004 in the free tropospheric CO background as measured at the Jungfraujoch, Switzerland, though with a significant excursion from this pattern when CO rose in the heatwave year of 2003. This strong overall multi-year fall in CO at Jungfraujoch contrasted with a much slower rate of decrease at Zugspitze, Germany^15^, where CO fell by −0.84±0.95 ppb/yr between 1991 and 2004. Moreover, both Petrenko *et al*.^5^ and Wang *et al*.^6^ report declining global CO in recent years.

**Figure 7:**
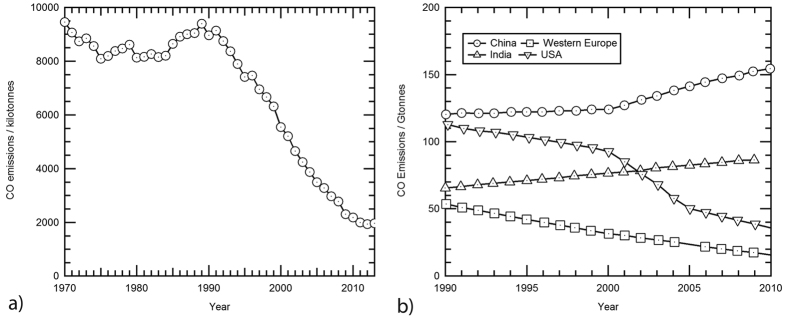
CO Emissions as reported in national and global emissions inventories. (**a**) UK National CO emissions inventory (totals), 1970–2013, summarised using data from the UK National Atmospheric Emissions Inventory^25^. UK emissions decreased by 76% between 1990 and 2010 compared to 62% for the EC27. (**b**) Comparison of CO emissions estimates 1990–2010: US, Western Europe, India and China using the ACCMP/MACCity estimates^28^.

CO emission inventories (Fig. 7) imply that CO emissions are being reduced both globally and also regionally in the European Union. Inventory data are collected “bottom-up”: i.e. they are based on estimates of emissions from various categories of CO sources – dominantly road traffic, residential, and other combustion sources. “Bottom-up” inventories are difficult to construct, as small errors in per-unit estimates of fluxes from components such as vehicles can lead to large quantitative errors when scaled up to the national inventory. Thus inventory emissions estimates are quoted to high precision and are very reproducible but may have low inherent accuracy. In contrast, ‘top-down’ estimates are accurate (the CO is actually measured), albeit within wide error margins, but have low precision as day-to-day fluctuations are wide and impair reproducibility.

Prior to 1992, reported UK emissions^25^ were roughly stable (Fig. 7a) fluctuating around 9 Mtonnes per year^26^ (note totals vary according to protocols for emissions definition). In 1991, the Road Vehicles (Construction and Use) Regulations introduced standards for petrol (gasoline) vehicle exhausts, enforced by annual testing. From 1992-5, there was a decline, then two years with similar emissions. Sustained strong decline set in after the implementation of the tougher National Air Quality Strategy in 1997, such that present emissions are less than a quarter of peak emissions^27^.

UK vehicle emissions testing regimes are rigorous, but a significant supporting factor in the success of the policy may have been the use of selective taxation to hasten the decline in the use of leaded petrol (gasoline) and the consequent improvement in catalyst performance. While the UK inventory (Fig. 7a) shows a rapid decline in emissions during the 1992–2009 period followed by a slow down in rate (in agreement with the clear slowdown in the decline observed in the EGH record from 2009 onwards, Table 1), the inventory estimates of European emissions^28^ show a more steady but slower decline from 1990–2010 (Fig. 7b). In contrast, the falling US emissions shows a period of rapid decline in the early 2000s followed by slow down,

### Hong Kong Comparison

Arguably the most serious current global CO problem in terms of measured mole fractions is in East Asia. Figure 7 shows the marked contrast between the decline in reported UK CO emissions and concurrent growth in Asian CO emissions inventories. India shows a steady rise during the 1990–2010 period, and the slow rate of increase in Chinese emissions speeds up significantly after 2000 (Fig. 7b).

To investigate this, Hong Kong, which has excellent CO records, was chosen as a case study. Hong Kong in many ways is comparable to London in that the populations are similar (7 to 8 million), as are the areas (1000–1600 km^2^). Both London and Hong Kong are wealthy cities located on the coastal margins of heavily populated industrial sub-continents. Both experience episodes of strong oceanic air-flow, as well as sustained periods of interior continental air. In London, prevailing wind is SW from the ocean but easterly continental air can arrive at any time of year. In Hong Kong the air-flow is generally bimodal – mainly from the South China Sea in the summer monsoon, and from the Chinese landmass in winter in the dry NE monsoon. Both cities have strong regulatory concerns, and effective local governance. Similarity extends to the transport vehicles, management, and, more significantly, planning and regulatory frameworks.

The Hong Kong Environmental Protection Department has maintained a public record of CO measurements since the late 1990s. Precision is not explicitly stated (see Supplementary Information S5), but data quality is clearly suitable for annual average use^29^. Two sites were chosen (Fig. S5): Tap Mun, close to the border with mainland China on the more rural eastern coast, and Causeway Bay, a heavily urbanized site on the north-east side of Hong Kong Island. Tap Mun in effect monitors regional background, including air arriving from China, while Causeway Bay samples the urban increment over the background.

**Figure 8:**
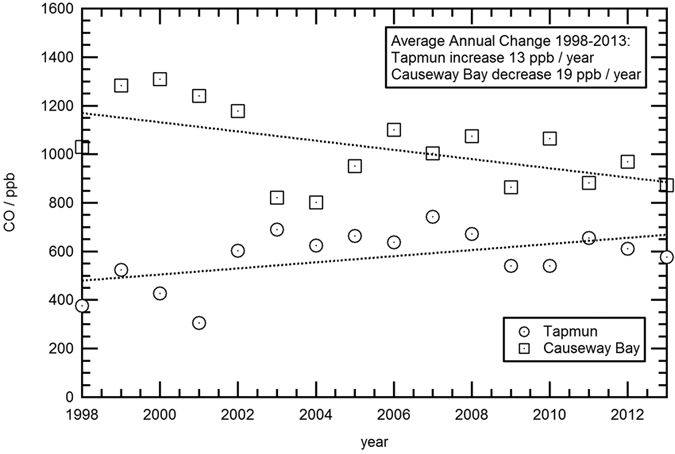
Hong Kong: annual averages of CO mixing ratios at Tap Mun (NE Hong Kong background) and Causeway Bay (east-central urban). Data from Hong Kong Environmental Protection Department^29^. Note that year-on-year meteorological changes can have large impact (for South China Sea Monsoon Index see http://web.lasg.ac.cn/staff/ljp/data-monsoon/SCSSMI.htm).

Figure 8 illustrates the yearly averages for the two stations, with CO converted from the data in the public record reported in μg/m^3^, assuming a 25 °C temperature. In 1998, the CO values at Causeway Bay averaged around 1000–1400 ppb, higher, but not greatly dissimilar to EGH during sustained easterly (continental) winds. By 2012, after sustained improvements, the Causeway Bay values had declined by more than 40% to an annual average of ~900 ppb. Note there was an instrumental upgrade in 2002, after which precision was better.

The Tap Mun regional background data show sustained CO growth to more than 700 ppb by 2007. This regional growth to 2007 is consistent with the findings of Granier *et al*.^30^ who record substantial (3% per yr) growth in Chinese CO over the post-2000 period. Since 2007 the Tap Mun record shows a possible slight decline to ~600 ppb. This is consistent with the findings of Worden *et al*.^7^ who observed a significant recent reduction in total column CO over China due to improved emission control on vehicles, and phasing out of residential coal stoves, as well as changes in industrial emissions. HYSPLIT back trajectory analysis indicates that CO elevations observed at the Hok Tsui regional background station in Hong Kong are sourced in the coastal regions of southern China and Eastern China within the previous 4-days^31^. Li and Liu^32^ also report reduced emission in the Beijing area, North China.

The data suggest (within the limits of instrumental precision) that the ‘Hong Kong increment’ – i.e. the CO increment due to local emissions in Hong Kong - has dropped from around 1000 ppb in the late 1990s to about 300 ppb in the post 2001 period. The London improvement is taking place against a regional reduction in Europe and with declining North American input to the Atlantic background that started 20 years earlier than the Chinese reductions.

## Discussion

CO is representative of many pollutants, and London of many northern European cities. While decadal variations in frequency of meteorological events, especially long-lived anticyclones such as those of summer 2003, may result in anomalous yearly averages, the sustained decline in CO suggests a significant and progressive underlying improvement in air quality in London. This observed decline is consistent with emission inventories and satellite observations, and comparable to the decline in other European cities^14^ but much higher than rates of decline observed at European continental background sites^15^. The cause of the London decline is almost certainly the strict controls on vehicle emissions introduced by the UK government, first in the 1991 Road Vehicles Regulations, and then the 1997 National Air Quality Strategy. Although background CO at Mace Head has been falling slightly since at least 1990^33^, there is no other obvious explanation of the sustained improvement in ambient CO in S.E. England. The UK legislation accompanied parallel moves across Western Europe in response to a European Union Directive, and the general improvement in London’s easterly air quality further confirms the betterment of air in the northern European source areas contributing to this flow.

The comparison with Hong Kong is instructive. Strong pollution control was introduced in the United Kingdom in 1997, and in Hong Kong in 1999. There the resemblance ends: the sub-continental regions differ in CO emissions history. Over the past 15 years the Egham area has reached near-oceanic background air quality, while urban Hong Kong has seen only limited decline in ambient winter CO. London benefits greatly from the wide emission reductions over NW Europe since 1990. Even in prolonged episodes of easterly air in winter, ambient CO mixing ratios at Egham now do not reach the excessive levels characteristic of the mid-late 1990s. However if it had not been for strenuous local emission control in the Hong Kong Special Administrative Region (SAR), the annual average CO mole fractions in Hong Kong could by now have been very much higher. Further improvements in Hong Kong during the sustained continental airflow of the winter NE Monsoon will depend on continued efforts to improve emission controls and thus air quality in mainland China^34^. If the recent reductions reported by Worden *et al*.^7^ apply also to south China, then there is a good prospect that the sustained two decades of improvement in London can be replicated in Chinese cities, such that air quality by, say, 2030 may return to near-background levels like London.

To conclude: effective improvement in ambient CO is indeed possible within a relatively short timeframe (a decade or less), but it needs both strong local action and co-ordinated regional policy.

## Methodology

### Analytical Methods

The analytical methodology is detailed in the Supplementary Information S1. In brief, CO measurements were made at Egham from late 1996 to the present. From 1997–2008, measurements were every 30 minutes by a Trace Analytical Reduction Gas Detector (RGD) instrument, precise to ±2 ppb, calibrated to NOAA standards from 2000. A Peak Laboratories Performer 1 analyser (PP1) was installed in January 2008, measuring every 5 minutes to ±1 ppb. From 2009 the data used in this study are from the PP1, though the overlap period when both instruments were operational extended to 2012 to confirm comparability of measurement (Fig. S1). Since 2014 a Los Gatos cavity-based analyser runs in parallel with the PP1.

### Data Analysis Methods

The data have been analysed extensively using the *Openair* software tools developed by King’s College, Univ. of London (http://www.openair-project.org/). Variations of atmospheric CO recorded at Egham by wind sector, wind speed and temperature have been assessed, as well as temporal variation at different scales: by time of day, day of the week and seasonal and annual cycles. This detailed assessment of local influences on CO will form part of a later manuscript.

## Additional Information

**How to cite this article**: Lowry, D. *et al*. Marked long-term decline in ambient CO mixing ratio in SE England, 1997–2014: evidence of policy success in improving air quality. *Sci. Rep.* **6**, 25661; doi: 10.1038/srep25661 (2016).

## Supplementary Material

Supplementary Information

## Figures and Tables

**Table 1:** Monthly averaged mixing ratios for CO measured at RHUL from 1997 to 2014.

Year\Month	Jan	Feb	Mar	Apr	May	Jun	Jul	Aug	Sep	Oct	Nov	Dec	Annual average*
1997	1090.7	442.8	591.6	564.3	467.1	343.2	369.2	531.0	489.7	795.3	630.8	658.5	581.2
1998	663.1	620.2	459.6	458.7	510.4	321.8	268.0	375.5	break	511.9	1017.9	632.2	530.8
1999	577.6	589.9	681.1	445.7	362.0	295.6	381.0	419.0	468.9	622.5	392.0	366.8	466.8
2000	463.4	365.2	413.6	379.5	303.7	237.3	229.0	202.6	236.6	307.1	421.7	591.9	346.0
2001	649.9	524.9	425.4	283.1	333.3	275.1	262.0	266.5	263.3	250.2	508.2	507.4	379.1
2002	394.4	259.7	394.2	259.7	385.6	317.8	230.0	191.3	198.9	232.2	311.3	393.5	297.4
2003	374.3	470.3	449.7	353.6	234.9	220.8	188.9	252.8	316.8	291.9	312.0	433.5	325.0
2004	273.6	309.2	375.1	247.8	225.1	166.8	164.4	184.9	186.1	229.5	306.9	416.7	257.2
2005	215.6	304.2	287.9	249.8	177.8	166.6	147.5	173.0	145.7	247.7	509.8	337.2	246.9
2006	389.3	325.6	273.4	239.4	207.6	207.6	184.4	135.6	175.9	214.2	280.5	284.8	243.2
2007	237.2	302.6	254.3	236.4	175.3	165.4	125.6	143.9	150.9	299.1	243.8	381.7	226.3
2008	206.5	353.7	198.4	220.8	224.0	155.1	133.7	129.5	190.2	172.9	200.1	254.9	203.3
2009	278.2	249.7	216.7	206.3	164.4	154.4	113.2	127.2	145.9	189.8	169.7	236.0	187.6
2010	189.9	243.0	203.8	205.6	169.8	154.2	124.0	127.4	166.9	178.8	246.2	266.4	198.0
2011	216.6	229.2	258.0	207.8	146.6	127.8	126.0	124.5	144.2	161.6	268.4	156.6	180.6
2012	221.4	235.2	227.9	172.8	173.3	126.4	124.0	143.4	165.7	198.2	230.1	215.5	186.2
2013	255.0	251.0	266.9	173.3	157.1	127.5	144.8	128.8	163.6	145.7	186.9	195.9	183.1
2014	188.9	157.2	226.5	190.4	157.1	126.9	119.8	133.0	187.8	152.9	278.1	180.0	174.9

*All the values are expressed as parts per billion (ppb).
