# Supplementary material for: Marked long-term decline in ambient CO mixing ratio in SE England, 1997–2014: evidence of policy success in improving air quality
Source: Sci Rep. 2016 May 23;6:25661. doi: 10.1038/srep25661 (PMC4876442; doi:10.1038/srep25661)
Supplement: Supplementary Information [file srep25661-s1.pdf]

SUPPLEMENTARY INFORMATION FOR

Marked long-term decline in ambient CO mixing ratio in SE England, 1997-2014: evidence of policy success in improving air quality

D. Lowry[1], M.E. Lanoisellé[1], R.E. Fisher[1], M. Martin[2], C.M.R. Fowler[1,3], J.L. France[4], I.Y. Hernandez-Paniagua[5], P. Novelli[6], S. Sriskantharajah[1], P. O'Brien[7], N.D. Rata[1], C.W. Holmes[8], Z.L. Fleming[9], K.C. Clemitshaw[1], G. Zazzeri[1], M. Pommier[10], C.A. McLinden[11] and E.G. Nisbet[1]**

*Corresponding authors

1. Dept. of Earth Sciences, Royal Holloway, Univ. of London, Egham, Surrey, TW20 0EX, United Kingdom.
2. Institute of Technology in Architecture, ETH, CH-8093 Hönggerberg, Zürich, Switzerland.
3. Darwin College, Cambridge, CB3 9EU, UK
4. School of Environmental Sciences, University of East Anglia, Norwich NR4 7TJ, UK
5. Escuela de Ingeniería y Ciencias, Tecnológico de Monterrey, Campus Monterrey, Av.Eugenio Garza Sada 2501, Monterrey, Nuevo León, México, C.P. 64849.
6. NOAA/ESRL Global Monitoring Division, 325 Broadway GMD-1, Boulder CO 80303, USA.
7. Irish Environmental Protection Agency, Richview, Clonskeagh Rd., Dublin 14, Ireland.
8. St.Richard's Church, Forge Lane, Hanworth, Middlesex, TW13 6UN, UK.
9. National Centre for Atmospheric Science (NCAS), Department of Chemistry, University of Leicester, Leicester LE1 7RH, UK
10. Sorbonne Universités, UPMC Univ. Paris 06, Université Versailles St-Quentin; UMR8190, CNRS/INSU, LATMOS-IPSL, Paris, France
11. Environment and Climate Change Canada, Air Quality Research Division, Toronto, Ontario MTH 5T4, Canada

S1. Methodology

The Royal Holloway Greenhouse Gas Laboratory (GGLES) measures CO_2, CH_4 and CO continuously, and $\delta^{13}C_{CH4}$ by spot sampling (though quasi-continuous measurement is also possible, particularly during major atmospheric inversion events). N_2O, H_2 and ^{222}Rn are also monitored. The air inlet is 2m above the highest point on the building, approximately 15m above ground level and roughly 30m above the London plain.

From Sept. 1996 until the end of 2012, CO measurements were made every 30-minutes by a Trace Analytical Reduction Gas Detector (RGD-2) instrument, coupled to a HP-5890 GC, using 2 $^{1}/_{8}$" OD columns packed in series: a Unibeads 1S and a Molecular Sieve 5A, with zero air as the carrier gas. The RGD-2 was calibrated twice-monthly from 2000 onwards against NOAA-CMDL calibrated air over the range 168 to 304 ppb CO to the WMO scale, with monthly averages recalibrated to the recent WMO X2014 scale. Precision averaged ± 2 ppb. Working standard cylinders were filled approximately every 3 months and calibrated against two NOAA-measured cylinders. These were measured half-hourly in the GC-RGD system, and if possible recalibrated against NOAA prior to exhaustion. Measurement of working standards post-2006 suggests drift in working standards may have been up to 5ppb over the 3 months of use. Intercomparison with other EU labs during the *Eurohydros* project (Engel, 2009) has shown that, like similar instruments of its vintage, the instrument is non-linear at high CO values (such as occur in polluted air). It may overestimate by as much as 30% in extreme events (>1800ppb). Three tanks filled and calibrated by MPI-Jena during

this project were used to maintain the scale over the 165 – 1170 ppb range, although doubt exists over the quality of the calibration for the high tank.

Since July 2007, measurement of CO has been by Peak Laboratories Performer 1 instrument (Reduced compound photometer), with similar columns and carrier gas to the RGD-2. Measurement is every 5 minutes with a precision of ±1 ppb, and with a rigorous manual calibration routine against laboratory standards, with current calibration against two NOAA-measured cylinders at 182 and 283 ppb on the WMO X2014 scale. A secondary standard was measured before each sample on the GC-RGD-2. On the PP1 the secondary standard is measured 4 to 6 times in a row, twice daily. During the 2008 period of instrument overlap there was very good R^2 agreement of 0.95 between the two instruments in the range from 80-600 ppb CO (see Fig. S1), suggesting no significant differences in linearity between the two instruments over this range. For WMO Intercomparison Round Robin results based on the PP1 analyser see Table S1.

Both the RGD-2 and PP1 instruments work on the same principles and are inherently non-linear and the response decreases with mixing ratio at a rate of approximately 3% per 100mV increase in output. No adjustments were made to the RGD-2 linearity during its use. The calibration equation obtained when the first 168.4 and 303.4 ppb NOAA cylinders were received in 2000 was used to back calibrate earlier data. Only 1 change was made to the sample inlet of the RGD-2 during the measurement period, when the original 1 cm^3 sample loop was changed to a 3.2 cm^3 sample loop in 2002 to give greater sensitivity at near background mixing ratios, as peaks over 2 ppm had disappeared from the record with the significant reduction in vehicle emissions. After this change the system was recalibrated against the NOAA cylinders, but no instrument linearity adjustments were made.

In the 1997-2002 period there were a number of major winter anticyclonic events in which very high CO was recorded at dawn. The extreme spikes were probably very locally sourced, as the wind velocities and inversion were very low. The non-linearity of the very high RGD-2 measurements, though brief and infrequent, may have introduced a very small bias to the annual time series. From 2002 until 2008 (when the RGD-2 was replaced) these extreme winter meteorological events were much less common or absent, and even when they did occur the general downward trend of regional CO emissions meant that the spikes were much less pronounced and thus less often encountered the range where the RGD-2 was significantly non-linear.

Engel, A. (2009) EUROHYDROS, A European Network for Atmospheric Hydrogen Observations and studies: Final Report, available on request from A. Engel (an.engel@iau.uni-frankfurt.de).

CO ppb	CA08186	CC86203	CA08182
NOAA	207.6 ± 1.4	173.3 ± 1.3	205.4 ± 1.4
RHUL	205.8 ± 0.9	172.3 ± 0.9	204.3 ± 0.7

Table S1: Results of blind Inter-Comparison of RHUL results with US NOAA standards. Intercomparison data from the 5th WMO Round Robin results in 2013.

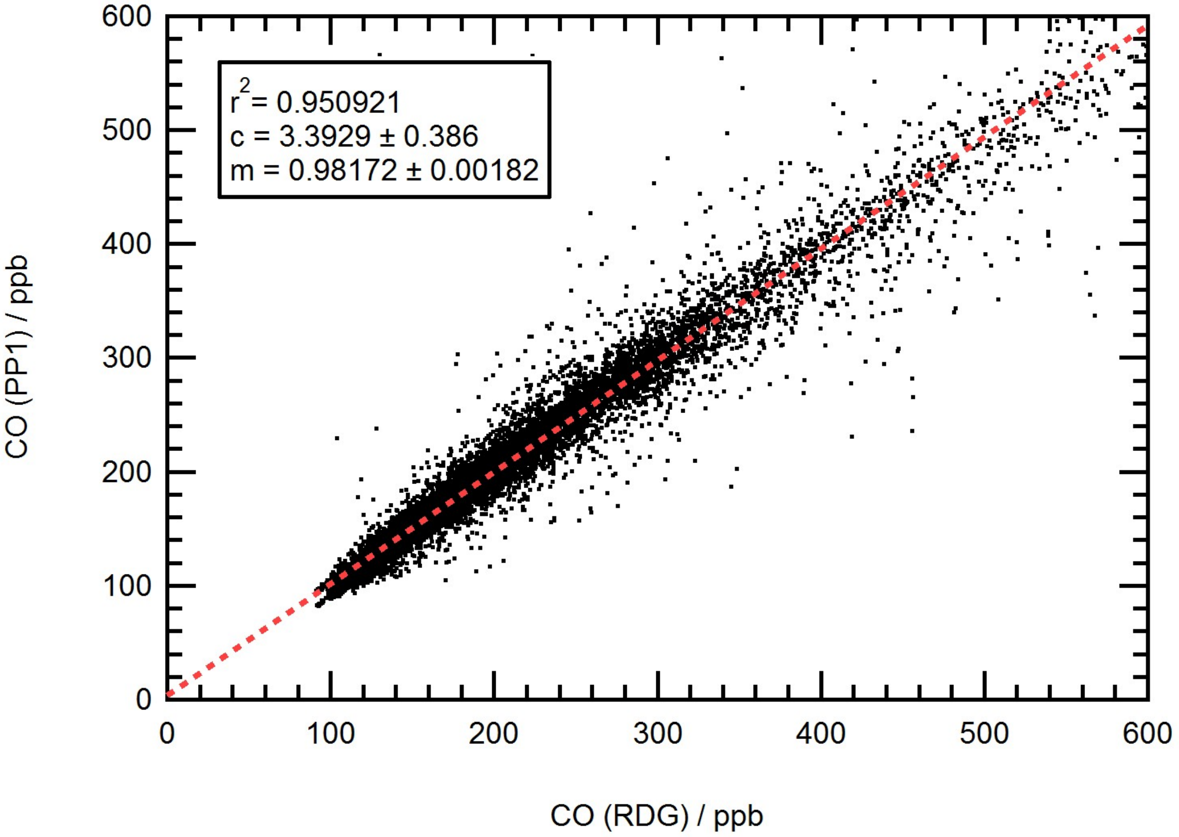

Figure S1 RHUL lab: Comparison cross plot of older RGD instrument and newer PP1 analyser for the overlap period of 2008, showing excellent agreement in near-simultaneous measurements during outside air monitoring.

S2. Comparison of CO (ppb) background records between Egham (RGD and PP1) and Mace Head (NOAA flasks) 1997 – 2014

S2.1 Calculating Egham monthly background

Egham CO data come from the RHUL RGD (Jan 1997- Dec 2008) and PP1 (Jan 2009 – Dec 2014) analysers, sampling air from 2m above the highest rooftop point at the Royal Holloway Earth Science Dept. CO values were selected from episodes when the wind was above a critical limit of wind speed of 1.0 m/s or greater. Additionally, to consider the effects of clean air from the Atlantic, only CO recorded with wind directions ranging from South to West were selected. Afterwards, the monthly minimum values of CO were calculated for all years. In each month, only CO values that are lower than or equal to this monthly minimum value plus 15 ppb (≤ monthly minimum + 15) were used. Finally, these selected CO values were averaged.

S2.2 Mace Head monthly background

Mace Head CO data were obtained from the US National Oceanic and Atmospheric Administration (NOAA) flask air measurements. The data are flagged and only those with status ‘accepted as background air sample’ or ‘has been measured and confirmed by other stations’ were chosen. Consequently, the number of data points varies between time intervals and there is no regular time interval between data points. NOAA data are accessible at: ftp://aftp.cmdl.noaa.gov/data/trace_gases/co/flask/surface/

S3. HYSPLIT clustering analysis

In all seasons the dominant source of air is seen to be from the West / South West, but with variations in proportions and trajectory length. January-March sees less than half the air masses coming from a W/SW direction compared with nearly two thirds of the air masses in July-September. In April there are notably mixed air masses, while October air masses frequently come from the continent to the SE.

Romensburg H C.(1984) *Cluster Analysis for Researchers.* Lifetime Learning Publications, Belmont, CA, 334p.

Stunder, B.J.B.(1996) An assessment of the quality of forecast trajectories. *J. Appl. Meteor.* **35**,1319-1331.

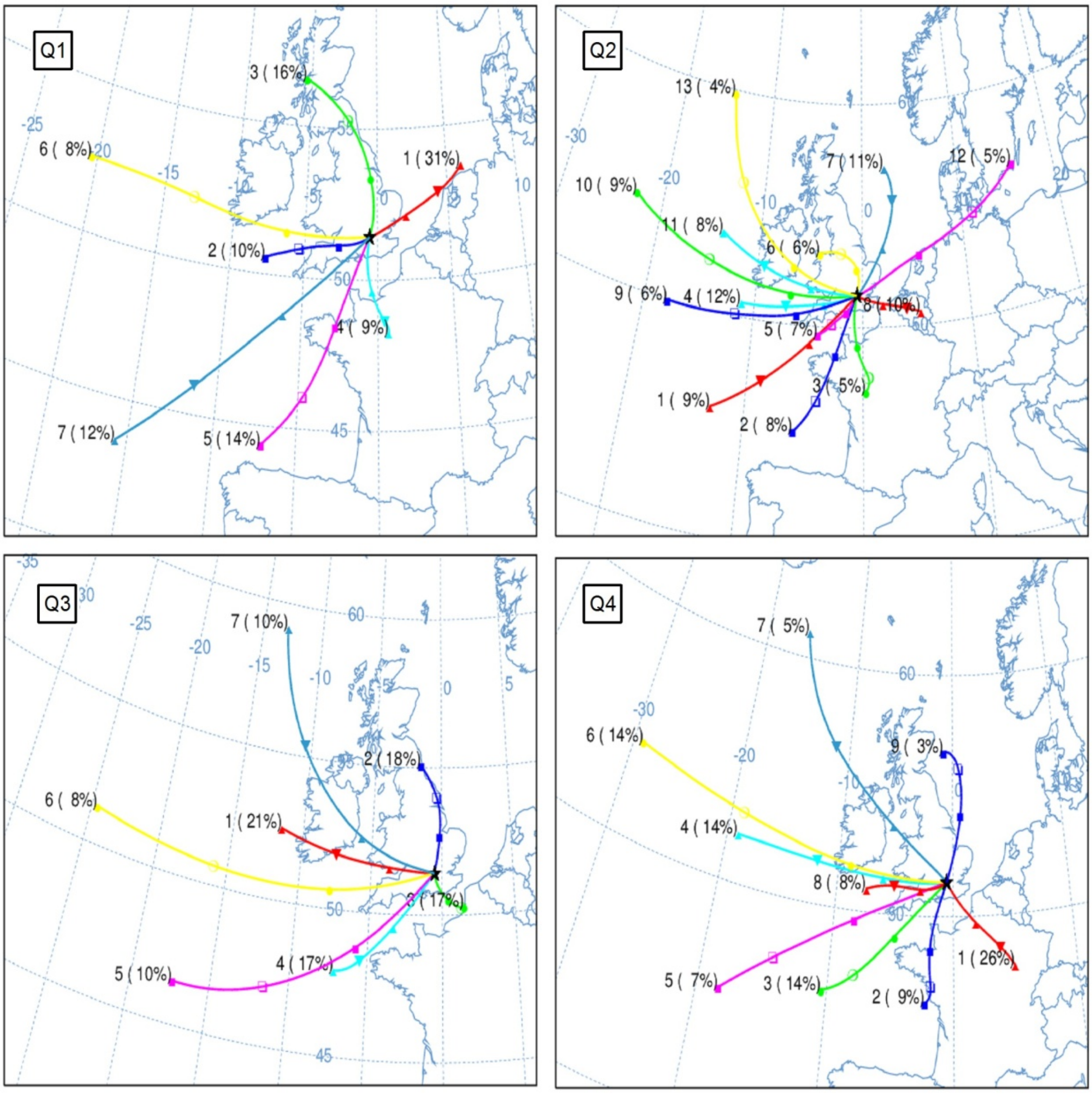

Figure S2. The clustering process in HYSPLIT uses Ward's method (Romensburg, 1984) and a detailed description is given in Stunder (1996). Only the first 36 hours of each back trajectory are considered when clustering the trajectories to give an indicative direction of input and to not over complicate the clustering procedure. Clusters were combined until the change in total spatial variance reached more than 30%. Four back trajectories per day were run for each quarter of the year (0000, 0600, 1200 and 1800 hours) with a start point of Egham. The background map for the HYSPLIT models is produced by ARL (Air Resources Laboratory) and is freely distributed with HYSPLIT (http://www.arl.noaa.gov/HYSPLIT.php). Trajectory maps are produced using archive data and can be freely redistributed (https://www.ready.noaa.gov/HYSPLIT_agreement.php).

S4. UK Emissions

United Kingdom CO emissions (Fig. S3) are reported in the UK National Atmospheric Emissions Inventory [25]. Carbon Monoxide data are available from the data selector page at: http://naei.defra.gov.uk/data/data-selector.

Figure S3 shows that the dominant factor behind changes in CO emitted in the UK is a reduction in road transportation emissions, together with smaller but similar underlying trends of decrease in residential emissions and from metal production. Interestingly the only significant increase in CO during the EDGAR analysis period is from electricity and heat production (Fig. S4). Note that the EDGAR database, while reflecting the UK national inventory, may differ in detail.

Hansard (1997) Official Report: edited verbatim report of proceedings of the UK Parliament.
http://www.publications.parliament.uk/pa/cm199697/cmhansrd/vo970204/debtext/70204-02.htm
http://cleanair.london/wp-content/uploads/CAL-294-NAQS-Research-Paper-1997-33.pdf

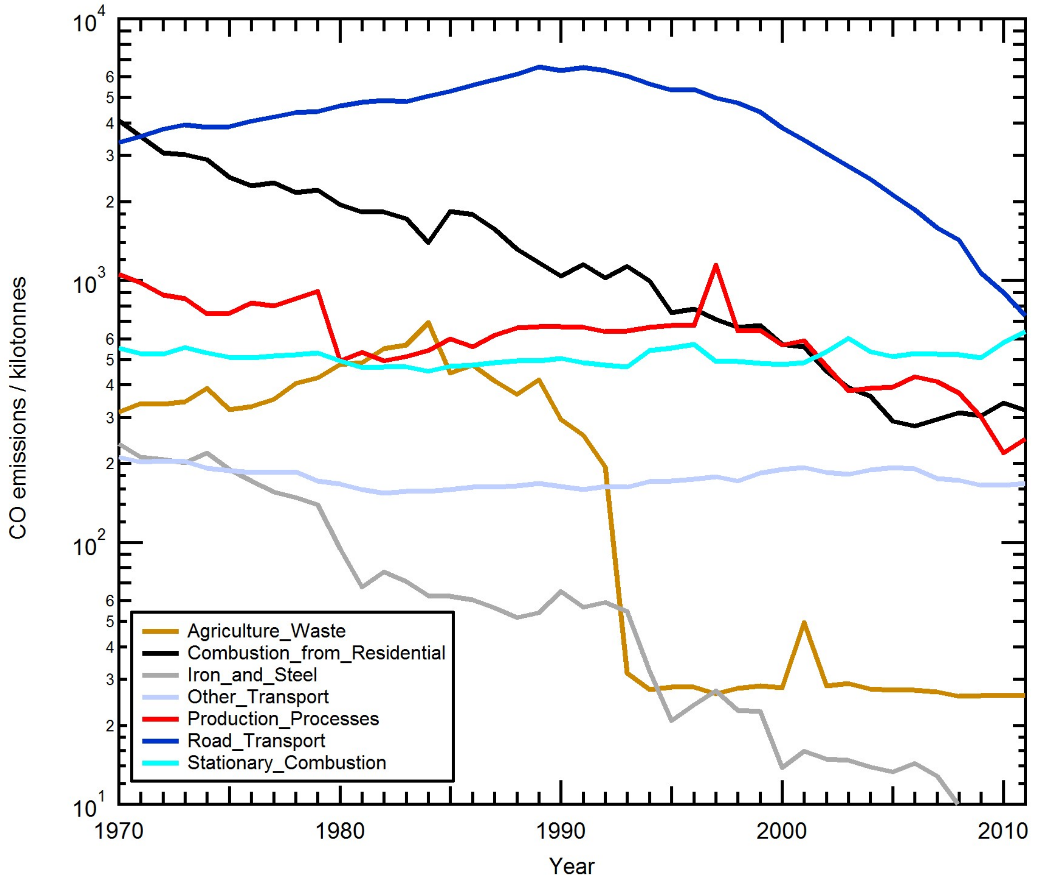

Fig. S3 UK CO emissions inventory by source type, 1970-2012. Data are replotted from data accessible in the public domain from http://naei.defra.gov.uk/data/data-selector?view=air-pollutants United Kingdom CO emissions are reported in the UK National Atmospheric Emissions Inventory [25,26,27]. The significant drop in Agricultural Waste in the early 1990s is due to the cessation of burning of agricultural residues.

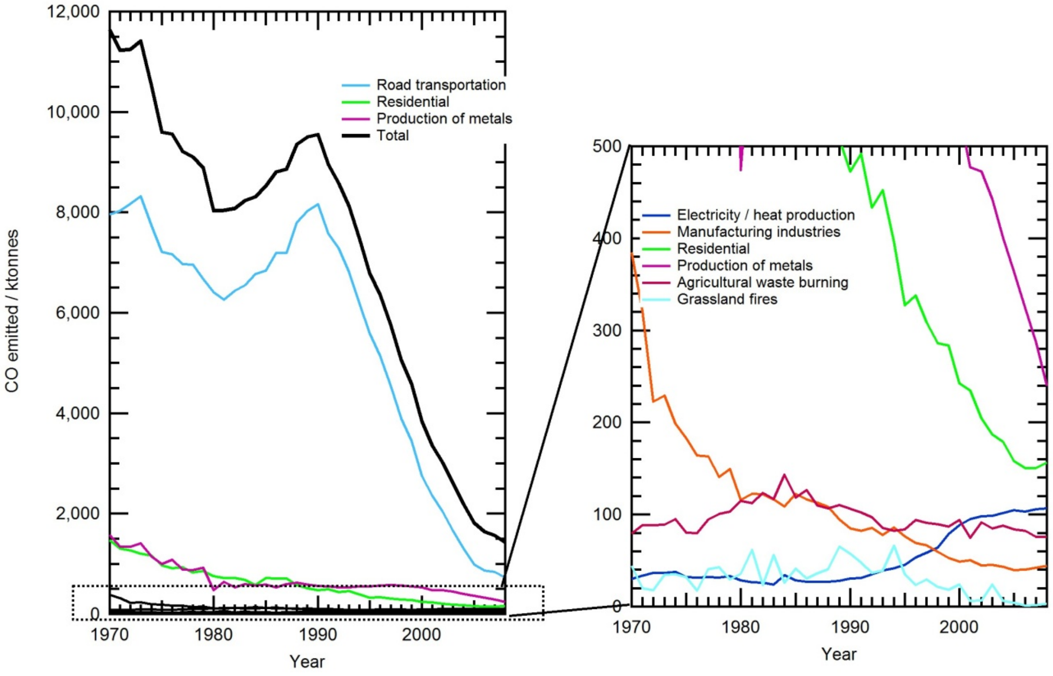

Figure S4 Sectoral distribution of UK CO emissions, 1970-2008, from EDGAR database.

S5. Hong Kong

Hong Kong CO data are publicly available from the Hong Kong Environmental Protection Department ([29] and Chow 2011). Data available on: http://epic.epd.gov.hk/ca/uid/airdata/p/1. Data were reported in values of 10μg/m^3 and were converted to ppb for a 25°C temperature, thus assuming that 10μg/m^3 x 0.873 x 10 gives mixing ratio in ppb.

Causeway Bay is an urban roadside site on the north shore of Hong Kong island. The air inlet is 3 m above ground level (8 m above mean sea level). Tap Mun is a rural background site on an island located in Mirs bay. The site lies 28 km NE of Causeway Bay. Measurements are validated by the Hong Kong Laboratory accreditation scheme.

Chow, C.F. (2011) Air Quality in Hong Kong 2011, Report number EPD/TR 2/12, Hong Kong Environmental Protection Department

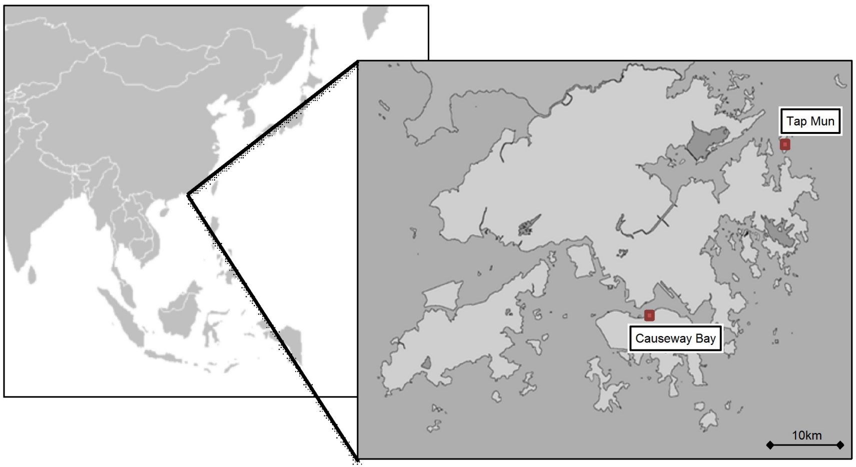

Figure S5 Location of measurement points in Hong Kong. Tap Mun samples winter regional background air arriving from the NE in the cold season, and Causeway Bay in the heavily urban core. Base maps for Hong Kong are adapted from those produced by www.mapsopensource.com under a Creative Commons Attribution 3.0 Unported License using Inkscape.
